# Profiling and characterization of a *longissimus dorsi* muscle microRNA dataset from an F_2_ Duroc × Pietrain pig resource population

**DOI:** 10.1016/j.gdata.2017.07.006

**Published:** 2017-07-05

**Authors:** Kaitlyn R. Daza, Juan P. Steibel, Deborah Velez-Irizarry, Nancy E. Raney, Ronald O. Bates, Catherine W. Ernst

**Affiliations:** a Department of Animal Science, Michigan State University, East Lansing, MI 48824, USA; b Department of Fisheries and Wildlife, Michigan State University, East Lansing, MI 48824, USA

**Keywords:** Pig, Skeletal muscle, Small RNA sequencing, miRNA

## Abstract

To elucidate the effects of microRNA (miRNA) regulation in skeletal muscle of adult pigs, miRNA expression profiling was performed with RNA extracted from *longissimus dorsi* (LD) muscle samples from 174 F_2_ pigs (~ 5.5 months of age) from a Duroc × Pietrain resource population. Total RNA was extracted from LD samples, and libraries were sequenced on an Illumina HiSeq 2500 platform in 1 × 50 bp format. After processing, 232,826,977 total reads were aligned to the *Sus scrofa* reference genome (v10.2.79), with 74.8% of total reads mapping successfully. The miRDeep2 software package was utilized to quantify annotated *Sus scrofa* mature miRNAs from miRBase (Release 21) and to predict candidate novel miRNA precursors. Among the retained 295 normalized mature miRNA expression profiles ssc­miR­1, ssc­miR­133a­3p, ssc­miR­378, ssc­miR­206, and ssc­miR­10b were the most abundant, all of which have previously been shown to be expressed in pig skeletal muscle. Additionally, 27 unique candidate novel miRNA precursors were identified exhibiting homologous sequence to annotated human miRNAs. The composition of classes of small RNA present in this dataset was also characterized; while the majority of unique expressed sequence tags were not annotated in any of the queried databases, the most abundantly expressed class of small RNA in this dataset was miRNAs. This data provides a resource to evaluate miRNA regulation of gene expression and effects on complex trait phenotypes in adult pig skeletal muscle. The raw sequencing data were deposited in the Sequence Read Archive, BioProject PRJNA363073.

**Table:** 

Specifications	
Organism/cell line/tissue	*Sus scrofa*/skeletal muscle
Sex	Male and female
Sequencer or array type	Illumina HiSeq 2500
Data format	Raw
Experimental factors	Pigs for transcriptional profiling selected from larger sample set based on phenotypes for loin muscle area and 10th rib backfat thickness
Experimental features	Small RNA sequencing using RNA extracted from skeletal muscle of 176 F_2_ Duroc × Pietrain pigs, and quantification and characterization of miRNAs
Consent	N/A
Sample source location	Michigan State University, East Lansing, MI, USA

## 1 Direct link to deposited data

The deposited sequencing data can be found here: https://www.ncbi.nlm.nih.gov/bioproject/PRJNA363073

## 2 Experimental design, materials and methods

### 2.1 Sample collection, RNA isolation and sequencing

A subset of 176 F_2_ pigs from 44 litters (two males and two females per litter) of the Michigan State University Duroc × Pietrain Pig Resource Population (MSUPRP) were selected for transcriptional profiling [1], [2], [3]. Samples of *longissimus dorsi* (LD) tissue were collected from each animal at slaughter and frozen at − 80 °C. Animal care and experimental protocols were approved by the Michigan State University All University Committee on Animal Use and Care (AUF# 09/03-114-00). Total RNA was isolated from LD samples using the miRNeasy Mini Kit (QIAGEN, California, USA), and small RNA library construction and sequencing was performed at the MSU Research Technology Support Facility. Samples were prepared for sequencing utilizing the Bioo Scientific NEXTFlex™ Small RNA Sequencing Kit (v2; Bioo Scientific, Austin, TX, USA). Libraries were barcoded and multiplexed for sequencing on the Illumina HiSeq 2500 platform (Illumina, Inc.; California, USA) in 50 bp, single-end format. Two libraries failed to produce acceptable sequencing output and were removed from further analysis, yielding 174 files of 50 nt short read sequences in fastq format (86 males, 88 females).

### 2.2 Analysis of sequencing data

The 3′ adaptor sequences were trimmed from raw short reads using cutadapt (cutadapt/1.4.1; [4]), and trimmed reads of length 26–38 nt were filtered for quality using FASTX toolkit (FASTX/0.0.14; http://hannonlab.cshl.edu/fastx_toolkit), removing any reads in which > 50% of the nucleotides had Phred score < 30. The selected sequence length retained an additional 4 nt on both the 5′ and 3′ ends of each read to accommodate the randomized adaptors ligated during library preparation, which allowed the identification of PCR duplicates. Distribution of sequence read lengths was assessed, and trimmed, filtered reads were collapsed using FASTX toolkit (FASTX/0.0.14; http://hannonlab.cshl.edu/fastx_toolkit) into unique expressed sequence tags containing a sequence identifier and the numerical count of each read (read count). PCR duplicates and random adaptor sequences were removed using the ShortRead package of R prior to genome alignment and quantification [5].

### 2.3 Characterization of small RNAs

To characterize classes of small RNA present in the dataset, unique expressed sequence tags underwent multiple queries utilizing BLAST + (v2.2.30) prior to read alignment and quantification of known miRNAs. Unique sequences expressed at least two times were included in this analysis. Sequences were sequentially queried against: the *Sus scrofa* mature miRNA miRBase database (Release 21) [6], the Ensembl *Sus scrofa* ncRNA database (Release 84), and the *Sus scrofa*, *Homo sapiens*, and *Mus musculus* Rfam databases (version 11) using blastn. The blastn-short parameter was implemented as it is optimal for queries of sequences < 50 nt, and an e-value threshold of 1 × 10^− 5^ was used to declare a significant hit. Results of each query were filtered as follows to retain unique sequence read hits: hits were retained that obtained 100% sequence identity, followed by at least 96% sequence identity, and finally hits with the maximum bitscore were retained. If multiple BLAST + hits remained for a given sequence, the hits most identical to the sequence were retained (based on percent identity). If multiple significant hits persisted with equal sequence identity, a single hit was retained for each sequence. This yielded the composition of classes of small RNA present in the small RNA sequencing dataset, based on both the unique expressed sequence tags and total read counts.

The miRDeep2 software package (v0.0.5) was used to align high-quality reads to the *Sus scrofa* reference genome (v10.2.79; Ensembl), and to quantify *Sus scrofa* mature microRNAs (miRNAs) obtained from miRBase (Release 21) [6], [7]. The average abundance of each mature pig miRNA was adjusted for differences in sequencing depth between libraries by converting the read counts to counts per million (cpm) with the edgeR package of R, incorporating TMM normalization factors [8]. miRNAs expressed at < 1 cpm in ≥ 44 libraries were removed from the dataset prior to calculation of the normalization factors.

miRDeep2 [7] was also utilized to predict candidate novel miRNAs. The software was provided the known *Sus scrof*a mature and precursor miRNA sequences, and the known *Homo sapiens* mature miRNA sequences from miRBase (Release 21) [6] to search for sequence homology for novel miRNA prediction. The resulting candidate novel precursors were filtered based on: the miRDeep2 score output by the algorithm (≥ 10), the estimated probability of the novel miRNA being a true positive (≥ 91 ± 2%), a significant Randfold *p*-value, and a minimum read count of 10 reads for both mature and star (complementary) sequences. Retained sequences were converted to DNA alphabet and FASTA format (ShortRead (R)) [5]. BLAST + (v2.2.30) was utilized to further characterize the candidate novel miRNA precursors by searching for sequence homology to the database of known human precursor and mature miRNAs in miRBase (Release 21) [6], utilizing a stringent e-value threshold of 1 × 10^− 5^ resulting in high-confidence homologous sequences.

All code used to analyze this dataset is publicly available and can be found at https://github.com/perrykai.

## 3 Data description

Determining underlying mechanisms controlling complex trait phenotypes such as growth, carcass composition and meat quality in pigs is important for achieving continued genetic improvement. One genetic mechanism involved in regulating these traits is the silencing of gene expression via miRNAs. Previous studies report that miRNAs exhibit dynamic expression in pig skeletal muscle across developmental stages, physiological states, and breeds [9], [10], [11], [12], [13], [14], [15]. Moreover, the signaling pathways enriched for genes that these miRNAs target are shown to be involved in myogenesis and muscle regeneration [11]. Due to the influential role of miRNAs on economically-important complex traits, it is necessary to continue characterizing miRNAs in pig skeletal muscle. Thus, the objective of this work was to profile and characterize the expression of miRNAs in LD muscle of market-age (approximately 5.5 months of age) F_2_ pigs from the MSUPRP [1], [2].

**Fig. 1:**
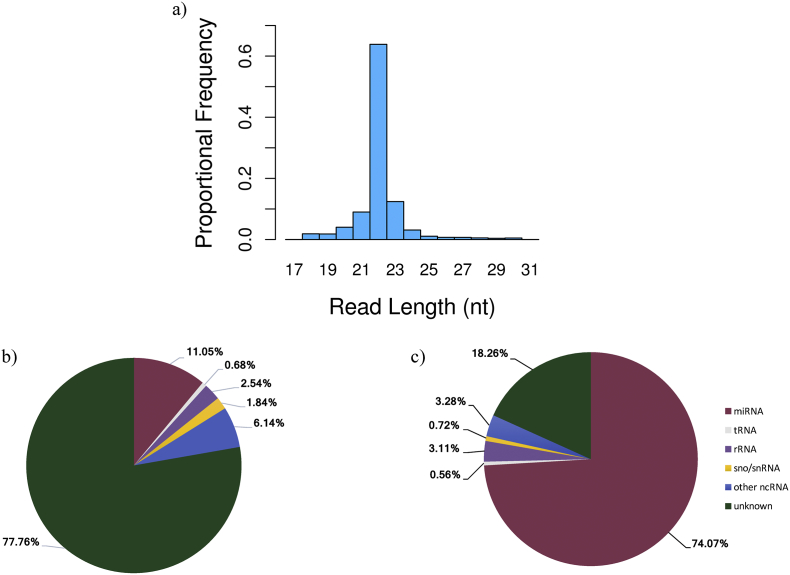
Characterization of small RNA sequencing data. a) Sequence read length distribution; composition of small RNA classes present in the small RNA sequencing dataset based on b) unique sequences, and c) total read counts.

Assessment of sequence read length distribution revealed 63.8% of the total reads to be 22 nt long, which is the common length of mature miRNAs. This indicates the success of small RNA sequencing at isolating miRNAs (Fig. 1a), and concurs with results in Xie et al. [12] where 43.7% of total small RNA sequencing reads were 22 nt in length. In total, 232,826,977 high-quality reads (mean 1,338,086 reads per library) were aligned to the *Sus scrofa* reference genome (v10.2.79; Ensembl), with 74.8% of reads successfully mapping. Moreover, 158,672,792 reads were quantified as miRNAs from the 174 samples, corresponding to 91.2% of the mapped reads.

After filtering for abundance across libraries, 295 miRNA expression profiles were obtained (Table S1). The five most abundant miRNAs represented 47.9% of the total cpm in the dataset including ssc-miR-1, ssc-miR-133a­3p, ssc­miR-378, ssc-miR-206, and ssc-miR-10b. These miRNAs have previously been identified in pig skeletal muscle, including Nielsen et al. [11], where ssc-miR-1 and ssc-miR-206 were the two most highly abundant miRNAs identified from sequencing LD samples from seven 1.5–2-year-old Danish Landrace/Yorkshire crossbred pigs. MiR-1, miR-206, and miR-133a are also considered the “myomiRs”, and have been well-characterized for their roles in mammalian skeletal muscle myogenesis and regeneration (for review, see [16]).

Results of the small RNA characterization analysis are shown in Fig. 1b, considering unique expressed sequence tags, and Fig. 1c, considering the total read count of each sequence. Most of the unique expressed sequence tags were not annotated in any of the five databases queried (“unknown”, 77.8%; Fig. 1b). Additionally, 11.1% of the unique expressed sequence tags (where each unique sequence was counted one time) were classified as miRNAs, and 6.1% of the unique expressed sequence tags consisted of other non-coding RNAs (Fig. 1b). When considering the sequences based on total read count, 74.1% of the sequences were identified as miRNAs, while 18.3% of the total reads were not annotated (Fig. 1c). These results show that miRNAs are the most abundant small RNA expressed in this dataset, and are consistent with results obtained in Xie et al. [12], where miRNAs were the most abundant class of small RNAs found in a pooled RNA sample from 16 pig tissues.

**Fig. S1:**
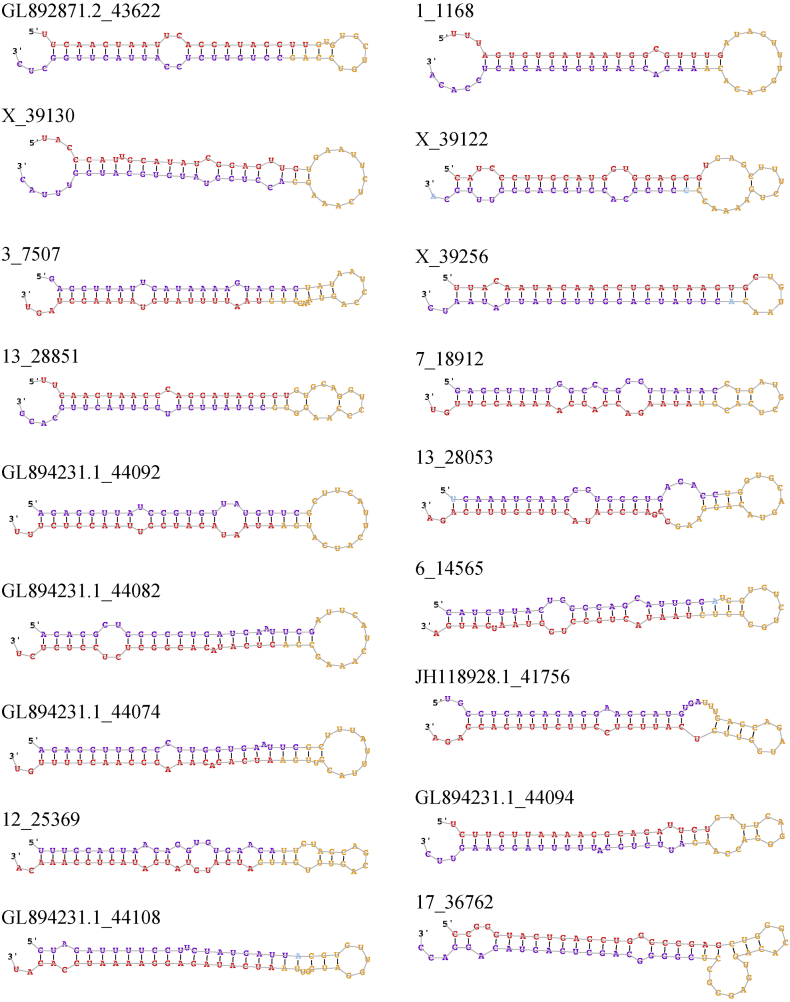

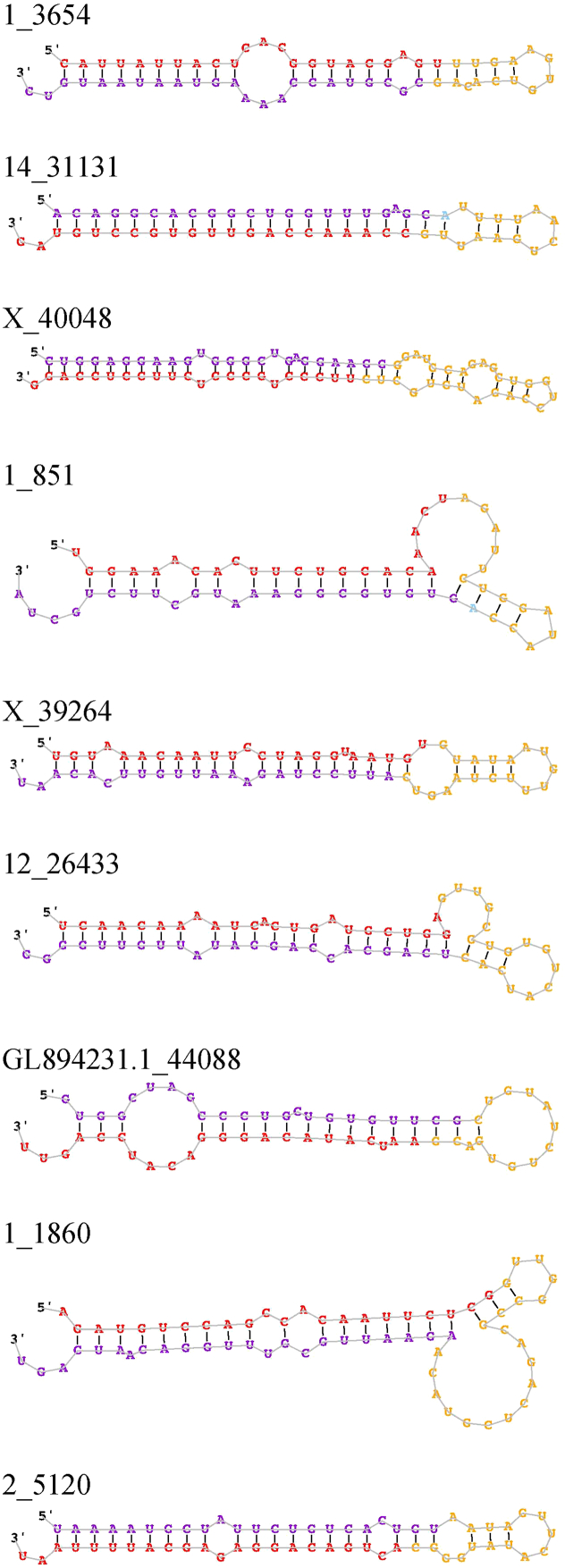
Secondary structures of candidate novel miRNAs. Sequences are color-coded as follows: red, mature sequence; yellow, loop sequence; purple, star strand sequence observed in small RNA sequencing data; light blue, expected star sequence based on Dicer/Drosha processing [7].

There were 132 candidate novel miRNAs predicted from miRDeep2 that passed filtering steps. After BLAST + query, there were 27 unique candidate novel miRNA precursors homologous to a human miRNA precursor (Table 1; additional annotation information in Table S2). Eight candidate novel precursors had more than one homologous human miRNA precursor, ranging from 2 to 6 hits per candidate novel sequence. The consensus secondary structures for the 27 unique candidate novel precursors predicted by miRDeep2 can be found in Fig. S1.

**Table 1:** Candidate novel miRNA precursors predicted from miRDeep2 and characterized with BLAST +.

Sequence ID^a^	miRDeep2 Score	Read Count	Predicted Mature Sequence^a^	Sequence Length	miRDeep2-predicted miRBase miRNA^a^	BLAST + matched precursor^b^	Percent Identical^b^	*E*-value^b^	Precursor Coordinate^a^
GL892871.2_43622	572,746.3	1,123,410	uucaaguaauucaggauagguu	22	hsa-miR-26a-5p	hsa-mir-26b	100.0	2.00E − 23	GL892871.2:56,019..56076:+
X_39130	136,780.7	268,282	uacccauugcauaucggaguug	22	hsa-miR-660-5p	hsa-mir-660	97.7	2.00E − 17	X:48,640,826..48640884:+
3_7507	57,547.6	112,871	uaauuuuauguauaagcuagu	21	hsa-miR-590-3p	hsa-mir-590	96.0	1.00E − 06	3:11,024,999..11025060:+
13_28851	40,621.2	79,667	uucaaguaacccaggauaggcu	22	hsa-miR-26a-5p	hsa-mir-26a-1	96.1	7.00E − 20	13:24,885,255..24885316:−
13_28851	40,621.2	79,667	uucaaguaacccaggauaggcu	22	hsa-miR-26a-5p	hsa-mir-26a-2	95.8	4.00E − 06	13:24,885,255..24885316:−
GL894231.1_44092	12,387.7	24,290	auaauacaugguuaaccucuuu	22	hsa-miR-655-3p	hsa-mir-655	100.0	7.00E − 17	GL894231.1:23,071..23131:−
GL894231.1_44082	6189.5	12,132	gucauacacggcucuccucucu	22	hsa-miR-485-3p	hsa-mir-485	100.0	3.00E − 25	GL894231.1:16,616..16675:−
GL894231.1_44074	6021.2	11,802	aucacacaaaggcaacuuuugu	22	hsa-miR-377-3p	hsa-mir-377	98.2	5.00E − 24	GL894231.1:10,061..10121:−
12_25369	4096.6	8028	aucauguaugauacugcaaaca	22	hsa-miR-6516-3p	hsa-mir-6516	98.0	5.00E − 21	12:4,273,970..4274033:+
GL894231.1_44108	3936.2	7714	aucauagaggaaaauccacau	21	hsa-miR-376a-3p	hsa-mir-376a-2	95.8	4.00E − 18	GL894231.1:31,685..31743:−
GL894231.1_44108	3936.2	7714	aucauagaggaaaauccacau	21	hsa-miR-376a-3p	hsa-mir-376a-1	85.2	2.00E − 07	GL894231.1:31,685..31743:−
1_1168	3418.9	6698	uuuagugugauaauggcguuug	22	hsa-miR-3591-5p	hsa-mir-3591	98.0	3.00E − 22	1:179,916,350..179916408:+
1_1168	3418.9	6698	uuuagugugauaauggcguuug	22	hsa-miR-3591-5p	hsa-mir-122	97.9	2.00E − 20	1:179,916,350..179916408:+
X_39122	3415.6	6691	caucccuugcaugguggagggu	22	hsa-miR-188-5p	hsa-mir-188	100.0	1.00E − 21	X:48,632,394..48632453:+
X_39256	1851.7	3631	uuacaauacaaccugauaagugc	23	–	hsa-mir-374a	96.1	6.00E − 20	X:67,530,381..67530433:+
X_39256	1851.7	3631	uuacaauacaaccugauaagugc	23	–	hsa-mir-374a	90.5	8.00E − 10	X:67,530,381..67530433:+
X_39256	1851.7	3631	uuacaauacaaccugauaagugc	23	–	hsa-mir-374c	88.9	3.00E − 09	X:67,530,381..67530433:+
X_39256	1851.7	3631	uuacaauacaaccugauaagugc	23	–	hsa-mir-374c	86.4	3.00E − 06	X:67,530,381..67530433:+
X_39256	1851.7	3631	uuacaauacaaccugauaagugc	23	–	hsa-mir-374b	88.9	3.00E − 09	X:67,530,381..67530433:+
X_39256	1851.7	3631	uuacaauacaaccugauaagugc	23	–	hsa-mir-374b	86.4	3.00E − 06	X:67,530,381..67530433:+
7_18912	1362.7	2664	auaagacgagcaaaaagcuugu	22	hsa-miR-208a-3p	hsa-mir-208a	100.0	3.00E − 25	7:81,043,856..81043913:−
13_28053	986.0	1925	gcgacccauacuugguuucaga	22	hsa-miR-551a	hsa-mir-551b	94.9	1.00E − 12	13:115,915,962..115916023:+
6_14565	642.0	1250	uaauacugccugguaaugauga	22	hsa-miR-200b-3p	hsa-mir-200b	95.0	2.00E − 13	6:58,064,309..58064367:+
JH118928.1_41756	527.0	1036	ucauucuccuucuuugaccaga	22	–	hsa-mir-6758	94.4	6.00E − 11	JH118928.1:123,819..123880:−
GL894231.1_44094	365.0	715	ucuuguuaaaaggcagauucu	21	–	hsa-mir-544a	97.6	6.00E − 17	GL894231.1:24,222..24280:−
17_36762	298.5	583	ccggguacugagcuggcccgag	22	–	hsa-mir-486-1	91.2	7.00E − 14	17:12,191,239..12191302:+
17_36762	298.5	583	ccggguacugagcuggcccgag	22	–	hsa-mir-486-2	91.1	3.00E − 13	17:12,191,239..12191302:+
17_36762	298.5	583	ccggguacugagcuggcccgag	22	–	hsa-mir-486-2	100.0	3.00E − 07	17:12,191,239..12191302:+
1_3654	244.3	470	cauuauuacucacgguacgagu	22	hsa-miR-126-5p	hsa-mir-126	100.0	7.00E − 23	1:313,734,674..313734733:−
14_31131	225.4	433	ccaaaccaguugugccuguag	21	hsa-miR-6715a-3p	hsa-mir-6715b	95.4	4.00E − 15	14:133,619,338..133619395:+
14_31131	225.4	433	ccaaaccaguugugccuguag	21	hsa-miR-6715a-3p	hsa-mir-6715a	93.5	2.00E − 14	14:133,619,338..133619395:+
X_40048	137.0	266	uucccugcccucuuccuccagg	22	–	hsa-mir-6894	89.6	8.00E − 11	X:51,475,450..51475523:−
1_851	130.1	248	uggaaacacuucugcacaaacu	22	hsa-miR-4261	hsa-mir-147b	95.9	1.00E − 18	1:140,919,000..140919059:+
X_39264	117.7	224	uguaaacaauuccuagguaaugu	23	hsa-miR-30a-5p	hsa-mir-384	88.4	6.00E − 08	X:69,515,840..69515903:+
12_26433	111.2	209	ucaacaaaaucacugaugcugga	23	hsa-miR-3065-5p	hsa-mir-338	94.7	5.00E − 21	12:1,507,090..1507153:−
12_26433	111.2	209	ucaacaaaaucacugaugcugga	23	hsa-miR-3065-5p	hsa-mir-3065	94.1	2.00E − 17	12:1,507,090..1507153:−
GL894231.1_44088	80.7	150	aaucauacagggacauccaguu	22	hsa-miR-154-3p	hsa-mir-487a	90.0	1.00E − 08	GL894231.1:20,450..20508:−
1_1860	61.0	111	agauguccagccacaauucucg	22	hsa-miR-219b-5p	hsa-mir-219b	100.0	3.00E − 22	1:302,806,862..302806927:+
1_1860	61.0	111	agauguccagccacaauucucg	22	hsa-miR-219b-5p	hsa-mir-219a-2	100.0	2.00E − 20	1:302,806,862..302806927:+
1_1860	61.0	111	agauguccagccacaauucucg	22	hsa-miR-219b-5p	hsa-mir-219a-1	100.0	3.00E − 07	1:302,806,862..302806927:+
2_5120	24.3	39	acugacaggagagcauuuuaau	22	hsa-miR-3660	hsa-mir-3660	94.8	1.00E − 12	2:100,272,647..100272707:+

a Results obtained from miRDeep2 algorithm.

b Results obtained from BLAST + query against *Homo sapiens* miRBase database.

In summary, 295 known *Sus scrofa* mature miRNA expression profiles were obtained from next-generation sequencing of LD RNA from 174 F_2_ Duroc × Pietrain pigs of the MSUPRP. Twenty-seven unique candidate novel pig miRNA precursors were predicted, and the composition of classes of small RNA present in the dataset were characterized to reveal that miRNAs were the most abundant small RNA class expressed in the skeletal muscle of these pigs. This work contributes to the further characterization of microRNAs in pig skeletal muscle, which will lead to a more complete picture of the genetic regulation of complex economically-important pig production traits.

The following are the supplementary data related to this article.

Table S1 *Sus scrofa* mature miRNA average abundance (cpm) in the 174 F_2_ Duroc × Pietrain Michigan State University resource population pigs.
Table S2 Candidate novel miRNA annotation information. Image 2

## Conflict of interest

The authors declare no conflicts of interest.
